# Supplementary material for: Gut bacterial sphingolipid production modulates dysregulated skin lipid homeostasis
Source: bioRxiv. 2024 Dec 30:2024.12.29.629238. Preprint. [Version 1] doi: 10.1101/2024.12.29.629238 (PMC11722302; doi:10.1101/2024.12.29.629238)
Supplement: Supplement 1 [file media-1.pdf]

SUPPLEMENTAL FIGURES & TABLES

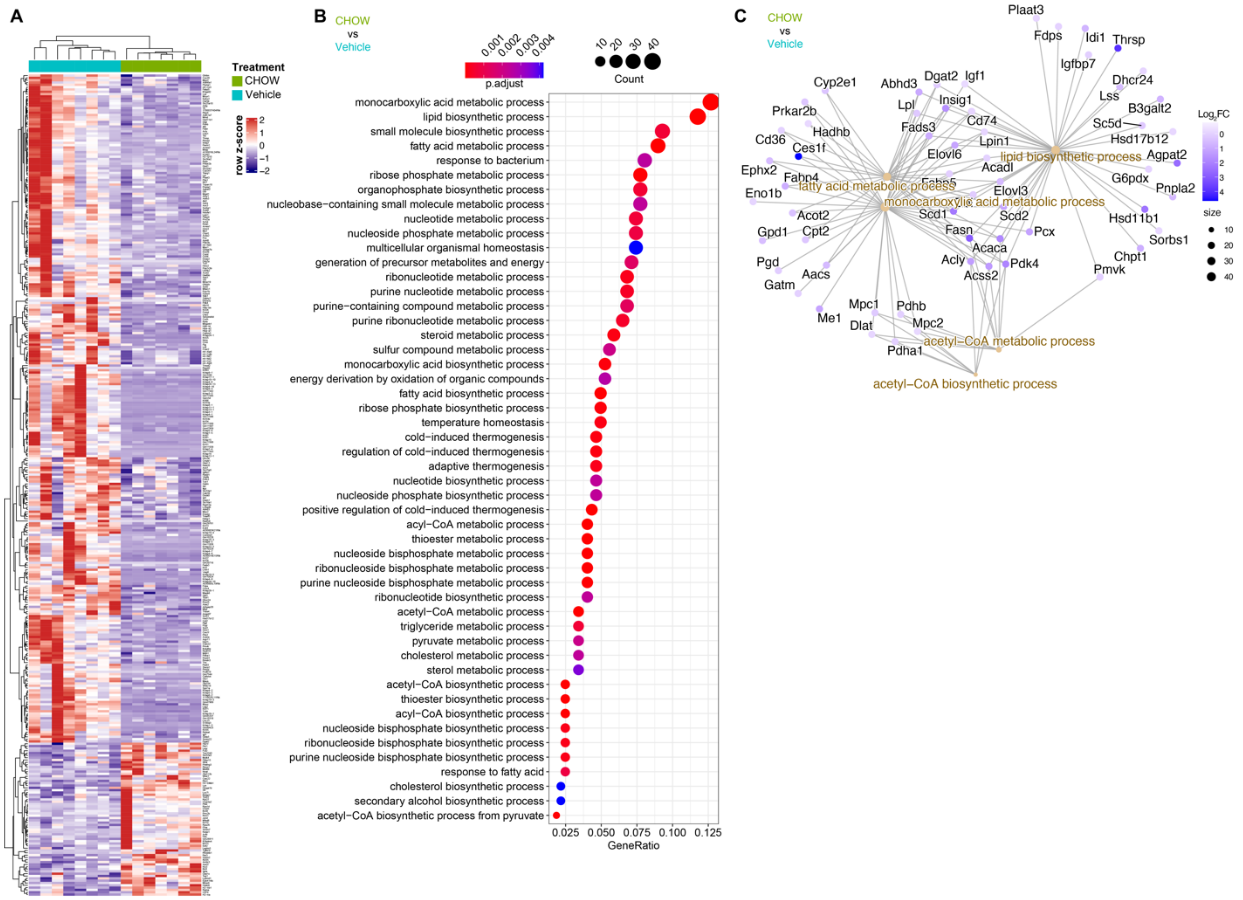

Supplemental Figure 1. (A) Heatmap displaying 354 significantly differentially expressed genes in the skin between mice fed the CHOW diet and mice fed the lipid-free diet (Vehicle). (B) The top 50 biological processes from gene ontology analysis (GO-BP) that are significantly differentially expressed between CHOW diet and lipid free-diet (Vehicle) fed mice at an adjusted p-value <0.05 ordered by enrichment values, calculated as the portion of genes under selection in the processes over the portion of genes significant in all GO-BPs. Points are color-filled by the adjusted p-value (p.adjust) and sized according to the gene numbers including in the corresponding GO-BP. (C) Gene lineage network plot shows the correlation among differentially expressed genes (DEGs) and GO-BP. Points are color-filled by the $\log_2$ fold change (Log_2FC) of genes compared between CHOW and Vehicle mice.

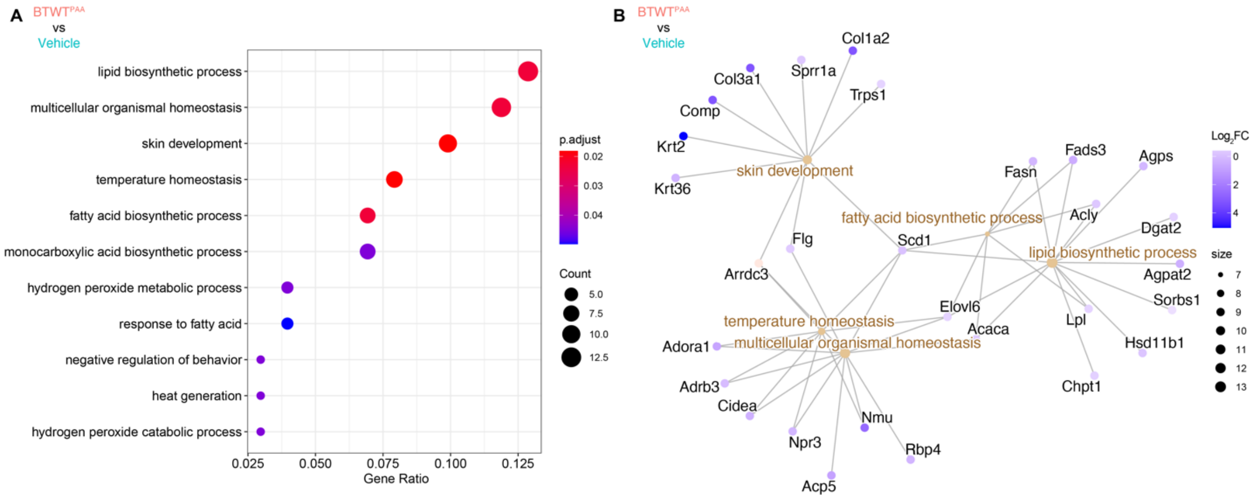

Supplemental Figure 2. Genes involved in lipid metabolism were enriched in the skin of mice that received sphingolipid-producing *B. thetaiotaomicron* (BTWT^{PAA}) compared to the mice fed solely lipid-free diet (Vehicle). (A) Biological processes in gene-ontology analysis (GO-BP) that are that are significant at the adjusted p-value (p.adjust), p <0.05, ordered by enrichment values, calculated as the portion of genes under selection in the processes over the portion of genes significant in all GO-BPs. Points are color-filled by p.adjust and sized according to the numbers of genes included in the corresponding GO-BP. (B) Gene lineage network plot shows the correlation among DEGs and GO-BP. Points are color-filled by the $\log_2$ fold change (Log_2FC) of genes compared between BTWT and Vehicle.

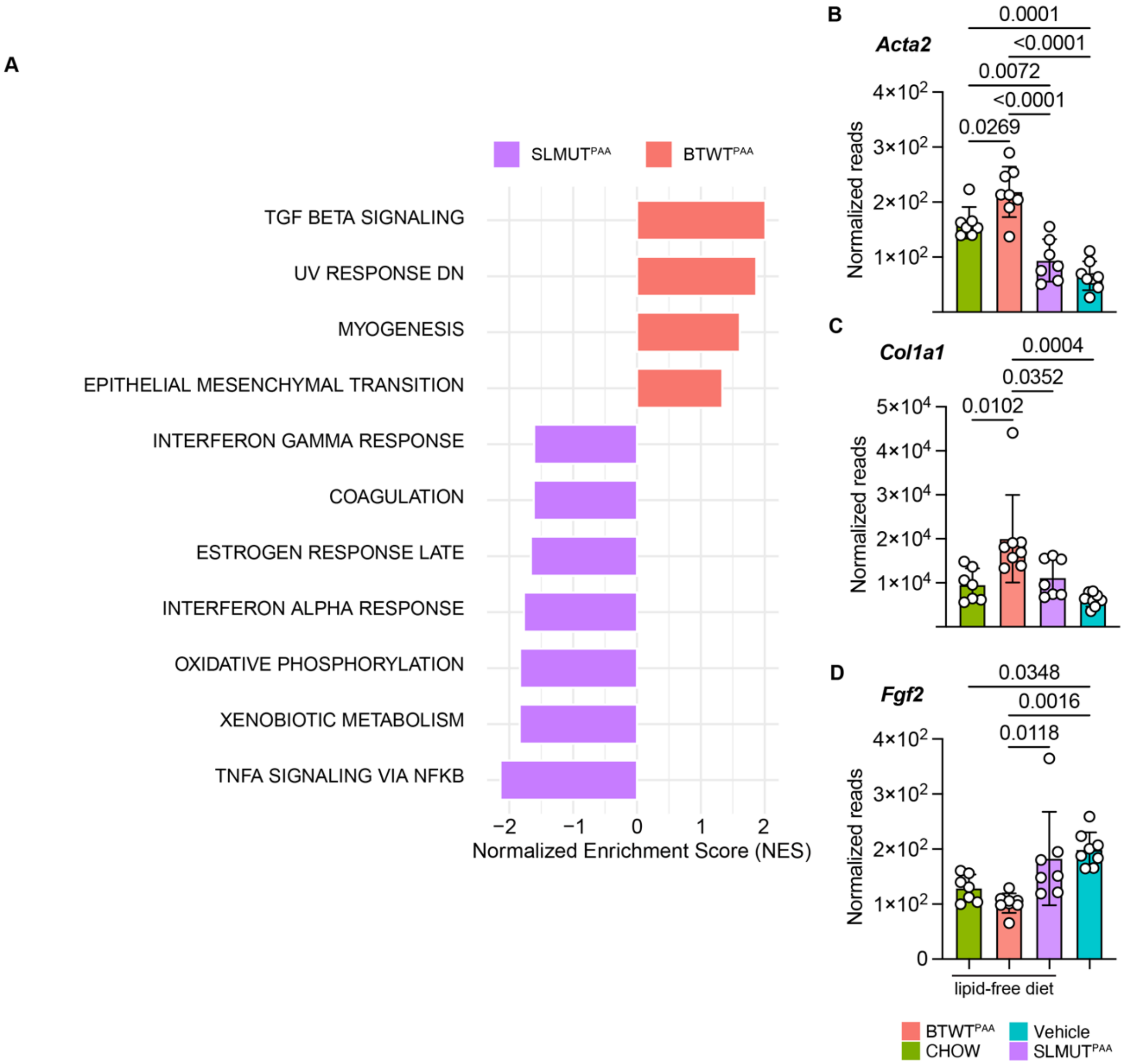

Supplemental Figure 3. Genes involved in TGF-β signaling and epithelial mesenchymal transition (EMT) were enriched in the skin of mice exposed to sphingolipid-competent versus sphingolipid-deficient *B. thetaiotaomicron.* Mice received either chow (CHOW) or a lipid-free diet; and mice fed with lipid-free diet were orally gavaged with either Vehicle, $BTWT^{PAA}$, or $SLMUT^{PAA}$. (n = 8 in either $BTWT^{PAA}$ or Vehicle; n = 7 in either CHOW or $SLMUT^{PAA}$) (A) Enriched Hallmark gene sets in either $BTWT^{PAA}$ or $SLMUT^{PAA}$. (B-D) Comparison of gene expression levels of (B) *Acta2* and (C) *Col1a1* in the TGF-β signaling pathway and (D) *Fgf2* in the antagonistic fibroblast growth factor 2 program.

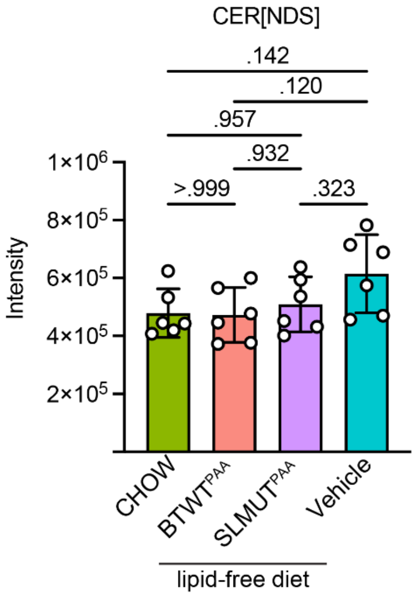

Supplemental Figure 4. No difference of the level of total CER[NDS] was found between groups. Mice received either chow (CHOW) or a lipid-free diet; and mice fed with lipid-free diet were orally gavaged with either a vehicle control (Vehicle), $BTWT^{PAA}$, or $SLMUT^{PAA}$. Total CER[NDS] levels in each group were calculated by summing up the sphingolipid species identified in this class. (n = 8 for both $BTWT^{PAA}$ and Vehicle; n = 7 for both CHOW and $SLMUT^{PAA}$)

Supplemental Table 1. SRM analysis

Ceramide Species	Precursor ions (Q1)		Product ion (Q3)	Collision energy (eV)
	**[M–H2O+H]+**	**$[M+H]^+$**		
**CER[NDS]**				
NDS (C16:0)		540.5	284.3	30
NDS (C20:0)		596.6	284.3	30
NDS (C24:0)		652.6	284.3	30
NDS (C26:0)		680.7	284.3	30
NDS (C28:0)		708.7	284.3	30
NDS (C30:0)		736.7	284.3	30
NDS (C32:0)		764.8	284.3	40
NDS (C34:0)		792.8	284.3	40
NDS (C36:0)		820.8	284.3	40
**CER[NS]**				
NS (C14:0)	492.4		264.3	30
NS (C18:0)	548.5		264.3	30
NS (C22:0)	604.6		264.3	30
NS (C26:0)	660.7		264.3	30
NS (C32:0)	744.8		264.3	40
NS (C36:0)	800.8		264.3	40
**CER[EOS]**				
EOS (C26:0)	938.9	956.9	264.3	30
EOS (C30:0)	995.0	1013.0	264.3	40
EOS (C34:0)	1051.1	1069.1	264.3	40
EOS (C26:1)	936.9	954.9	264.3	30
EOS (C30:1)	993.0	1011.0	264.3	40
EOS (C34:1)	1041.1	1067.1	264.3	40
**CER[OS]**				
OS/P-OS (C30:0)	732.7	750.7	264.3	30
OS/P-OS (C32:0)	760.8	778.8	264.3	40
OS/P-OS (C34:0)	788.8	806.8	264.3	40
